# Acanthosis Nigricans Presenting as Skin Tags: A Case Report

**DOI:** 10.7759/cureus.33706

**Published:** 2023-01-12

**Authors:** Ibrahim Alshareef, Sultan Assiri, Ashwaq K Alosaimi, Omar S Alhothali, Rola R Alsulami, Shahad Alkidaiwi, Khalid Al Hawsawi

**Affiliations:** 1 Dermatology, King Saud bin Abdulaziz University for Health Sciences College of Medicine, Jeddah, SAU; 2 Dermatology, King Abdulaziz Hospital, Makkah, SAU; 3 Medicine, Umm Al Qura University, Makkah, SAU

**Keywords:** warts, skin tags, dm1, an, acanthosis nigricans

## Abstract

Acanthosis nigricans (AN) is a common chronic disorder that is characterized by velvety-like, hyperpigmented, hyperkeratotic plaques, typically in intertriginous areas. However, atypical presentations have been reported. Here we present a five-year-old boy presented with a one-year history of asymptomatic slowly progressing skin lesions. He is a known case of type 1 diabetes mellites on insulin treatment, otherwise healthy. The review of systems was unremarkable. No similar case was found in the family. Skin examination revealed multiple tiny non-scaly brownish papules on the medial aspects of the upper thighs, bilaterally. Differential diagnosis included skin tags, viral warts, and dermatosis papulose nigra (DPN). Dermoscopic findings revealed a velvety-like appearance on the papules and the normal skin surrounding the papules. A 2-mm punch skin biopsy of the papule revealed papillomatosis of the epidermis, and the granular layer was normal. The dermis was normal. On the basis of the above clinicopathological findings, specifically the velvety texture of the normal skin surrounding the papules, the patient was diagnosed with ANs. The parent was reassured, and we started the patient on daily tretinoin cream.

## Introduction

Acanthosis nigricans (AN) is a chronic dermatosis that is characterized by velvety-like, brownish-black, hyperkeratotic plaques, typically in intertriginous areas. The neck is the most frequently affected area, followed by axillae; however, any area can be affected including eyelids, lips, vulva, mucosal surfaces, dorsum of hands, and flex areas in the groin, knees, and elbows [1]. It is associated with obesity, insulin resistance including type 2 diabetes, metabolic syndrome, polycystic ovary syndrome, and malignancy [1]. Early detection of these disorders is essential to prevent disease progression. AN is much more common in black people, especially African Americans. The prevalence of the disorder in whites is less than 1%. Latinos are more affected than whites [2]. The diagnosis of AN is made clinically. The lesions are characterized by symmetrical hyperpigmented, hyperkeratotic, and verrucous plaques. Hyperpigmentation appears to be due to thickening and hyperkeratosis rather than melanin excess [3]. We report an unusual case of AN, which presented as multiple tiny non-scaly brownish papules over the medial aspects of the upper thighs.

## Case presentation

**Figure 1:**
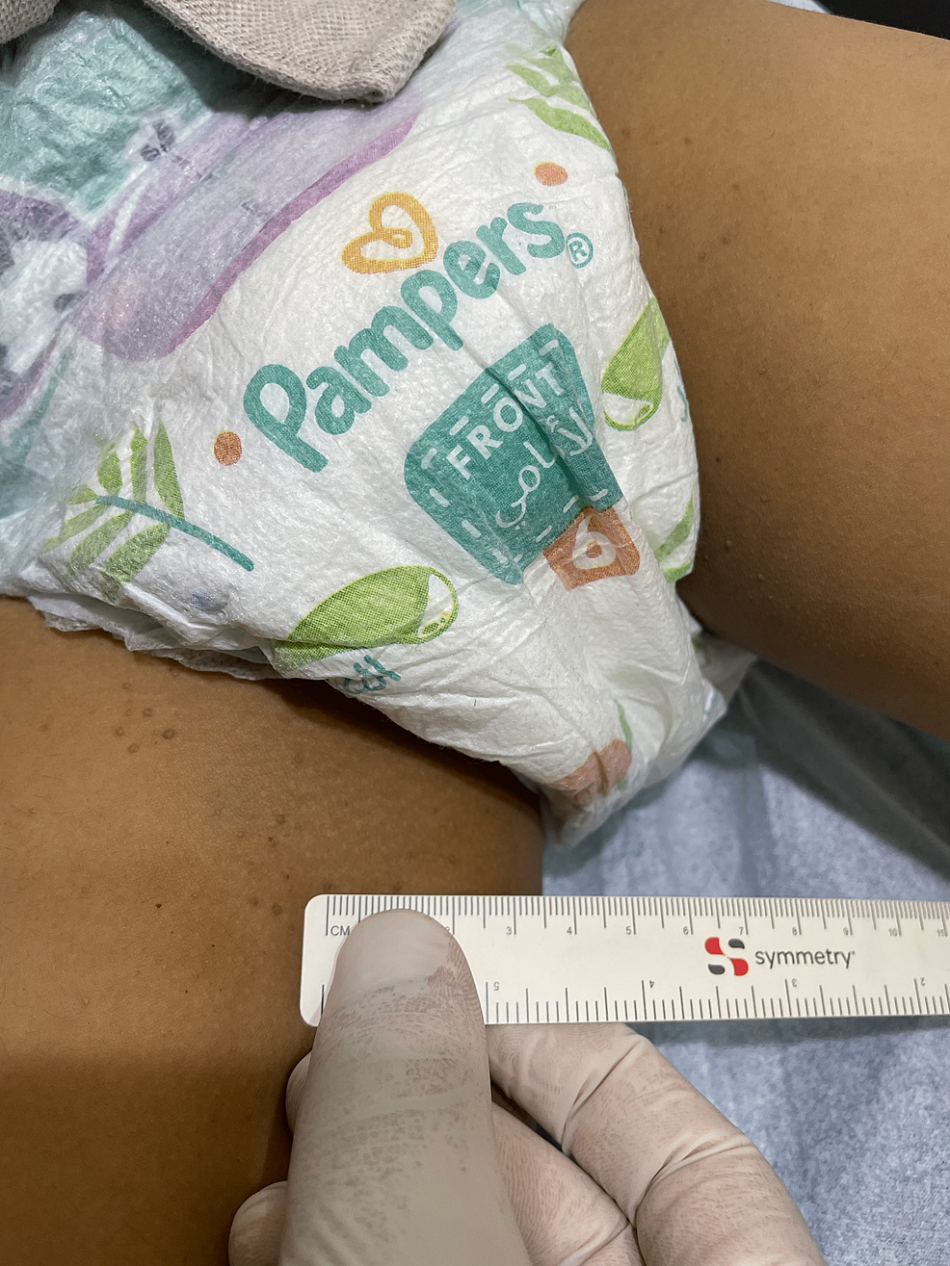
Skin examination The image shows the multiple tiny non-scaly brownish papules over the medial aspects of the upper thighs, bilaterally.

A five-year-old boy presented with a one-year history of asymptomatic slowly progressing skin lesions. He is a known case of type 1 diabetes mellites on insulin treatment, otherwise healthy. The review of systems was unremarkable. No similar case was found in the family. Skin examination revealed multiple tiny non-scaly brownish papules on the medial aspects of the upper thighs, bilaterally (Figure 1).

**Figure 2:**
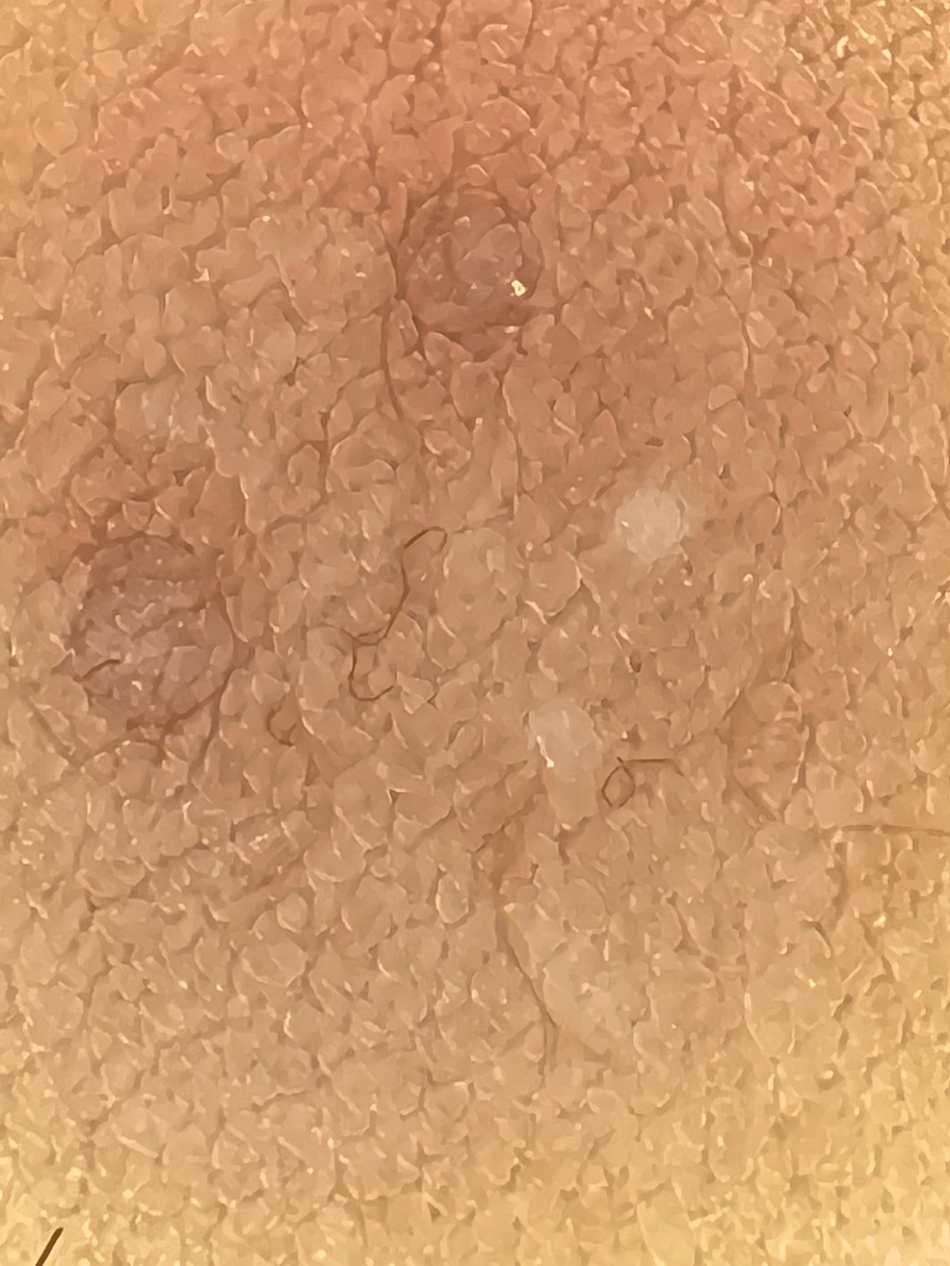
Dermoscopy The image shows the velvety-like appearance on the papules and the normal skin surrounding the papules.

Differential diagnosis included skin tags, viral warts, and dermatosis papulose nigra (DPN). Dermoscopic findings revealed a velvety-like appearance on the papules and the normal skin surrounding the papules (Figure 2).

**Figure 3:**
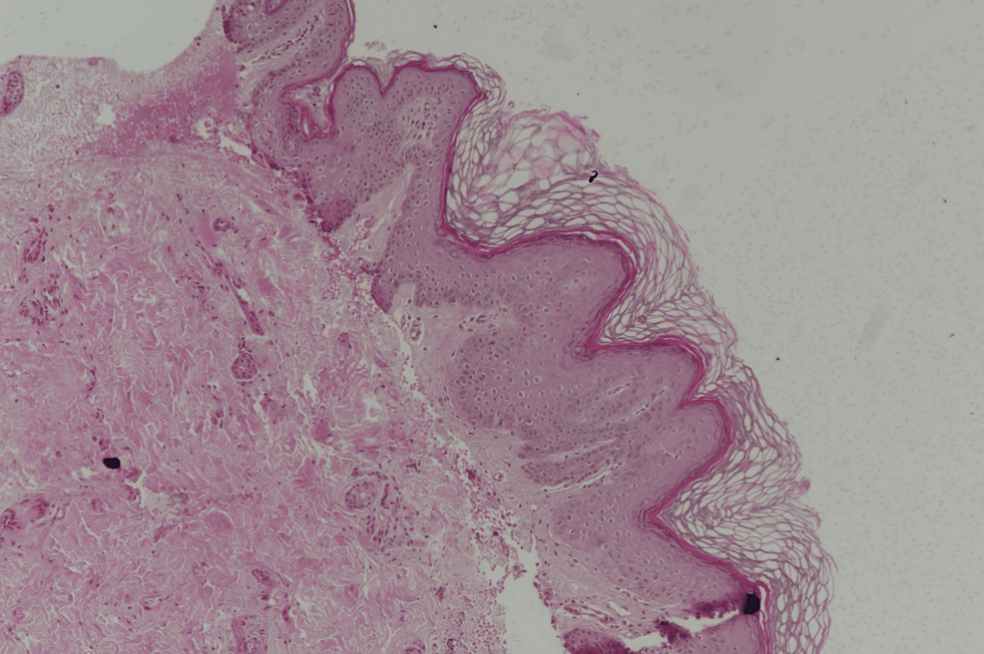
Pathology A 2-mm punch skin biopsy of the papule revealed papillomatosis of the epidermis; the granular layer and the dermis were normal (hematoxylin & eosin stain; original magnification, x20).

A 2-mm punch skin biopsy of the papule revealed papillomatosis of the epidermis, and the granular layer was normal. The dermis was normal (Figure 3).

On the basis of the above clinicopathological findings, specifically the velvety texture of the normal skin surrounding the papules, the patient was diagnosed with AN. The parent was reassured, and we started the patient on topical tretinoin cream 0.025% daily at night.

## Discussion

AN is a common dermatologic condition that typically presents as symmetrical hyperpigmented hyperkeratotic plaques. It is usually located in the intertriginous areas including axillae, neck, inframammary, and groin [4]. Other areas that can be involved include face, palmoplantar region, breast areola, and lip corners [5,6]. A generalized form without the presence of endocrine disease or internal malignancies has been reported [7]. Malignant AN presents as a paraneoplastic skin syndrome that is usually associated with gastric adenocarcinoma and can be the only initial sign of malignancy [8]. The differential diagnoses in our case include skin tags, viral warts, and DPN. However, the presence of grouped papules that are localized to medial aspects of the upper thighs bilaterally is unusual for skin tags, DPN, and warts. Dermatoscopy was very helpful in reaching the diagnosis in our case as it showed a velvety-like appearance of the normal skin surrounding the papules. This last feature is characteristic of AN. The skin biopsy confirmed the diagnosis of AN, which showed papillomatosis.

Papillomatosis refers to the projection of dermal papillae above the surface of the skin, resulting in an irregular undulating configuration of the epidermis. Papillomatosis can be seen in DPN and warts but not skin tags. However, other histopathological features like abnormal granular layer that is typical for warts and basaloid keratinocytes that are typical for DPN were all absent in our patient. Moreover, the velvety-like appearance of the normal skin surrounding the papules confirms the diagnosis of AN. There is not enough data supporting the effectiveness of different treatment modalities in the treatment of AN as most data are from case reports and physicians' experience. However, improvement was noted and reported with topical agents such as topical retinoids, keratolytics, and vitamin D analogs for localized lesions [9]. Generalized extensive lesions are usually treated by systemic retinoids such as isotretinoin and acitretin [10]. As our patient had only a few lesions, he was started on daily tretinoin cream.

## Conclusions

AN is a common disorder that can present at any age. However, it is less commonly seen in children than in adults. AN in non-obese people is associated with underlying systemic defects such as diabetes mellitus type 1 in our patient. Dermatologists should keep this atypical clinical presentation of AN in mind when seeing a patient with skin tag-like papules that are localized to one area. Hence, a thorough history and physical examination should be performed in addition to dermatoscopy and histopathology for atypical presentations.
